# Oral mycobiota and pancreatic ductal adenocarcinoma

**DOI:** 10.1186/s12885-022-10329-5

**Published:** 2022-12-02

**Authors:** Ailin Wei, Huiling Zhao, Xue Cong, Linyao Wang, Yiyang Chen, Juxiang Gou, Ziyi Hu, Xiuying Hu, Yali Tian, Ka Li, Yufeng Deng, Haojiang Zuo, Mei Rosemary Fu

**Affiliations:** 1 Guang’an People’s Hospital, Sichuan Province, Guang’an, 638001 China; 2 West China School of Nursing/Department of Otolaryngology-Head and Neck Surgery, West China Hospital, Sichuan University, Sichuan Province, Chengdu, 610041 China; 3 West China School of Public Health/West China Fourth Hospital, Sichuan University, No. 16, Section 3, Renmin South Road, Wuhou District, Chengdu, 610041 Sichuan China; 4 West China School of Nursing/West China Hospital, Sichuan University, Sichuan Province, Chengdu, 610041 China; 5 Rutgers University, School of Nursing–Camden, 530 Federal Street, Camden, NJ 08102 USA

**Keywords:** Pancreatic ductal adenocarcinoma (PDAC), Oral mycobiota, Biomarkers

## Abstract

Early detection of pancreatic ductal adenocarcinoma (PDAC) is essential for survival. Preliminary research demonstrated significant associations between structural alternation of mycobiota and PDAC. In this study, we investigated the associations between oral mycobiota and PDAC. We further explored mycobiota biomarkers for PDAC detection. We enrolled 34 PDAC patients and 35 matched healthy controls from West China hospital in Southwest China. Demographic data, clinical information, and salivary samples were collected. Mycobiota characteristics were defined using Internal Transcribed Spacer (ITS) ribosomal RNA sequencing. We found that the PDAC patients had significant increase in fungal abundance (*P* < 0.001) and significant decrease in fungal diversity (*P* < 0.001) in comparison to the healthy controls. A higher abundance of *Basidiomycota* and *Unclassifed_p_Ascomycota* was associated with an increased risk of PDAC. With each increase of abundance of *g__unclassified_k__Fungi* and *g__unclassified_p__Ascomycota* in PDAC patients, the risk of pancreatic cancer increased by 1.359 odds and 1.260 odds, respectively. *Aspergillus* (AUC = 0.983, 95% CI 0.951–1.000) and *Cladosporium* (AUC = 0.969, 95% CI 0.921–1.000) achieved high classification powers to distinguish PDAC patients from the healthy controls. The rapid, inexpensive tests of ITS1 sequencing of mycobiota and PCR detection of potential fungal biomarkers make it promising for the clinical practice to use oral microbes for PDAC early detection and prevention. Results of our study provide evidence that salivary mycobiota may provide insights into cancer risk, prevention, and detection.

**Supplementary Information:**

The online version contains supplementary material available at 10.1186/s12885-022-10329-5.

## Background

Research continues providing evidence on the significant associations between oral microbiota and cancers [1]. The oral microbes contain over 700 types of fungi, bacteria, and viruses [2]. Mycobiota, a group of all fungi, represents a small proportion of human microbiome, yet the potential effects of mycobiota on cancer may provide insights into the role of mycobiota in cancer risk, prevention, detection, and treatment. Preliminary research demonstrated significant associations between structural alternation of mycobiota and pancreatic ductal adenocarcinoma (PDAC) [3], colorectal carcinoma [4], and head and neck cancer carcinoma [5].

PDAC is the most prevalent type of pancreatic neoplasm that remains a deadly disease with about 5% of five-year survival rate [6, 7]. Early detection and diagnosis are essential for effective surgery treatment that improves cancer survival [8, 9]. Nevertheless, challenges remain for early PDAC detection because patients are usually asymptomatic at early disease stage and accuracy of current available detection methods are limited [10]. Serum Carbohydrate antigen 19–9 (CA19–9) as a biomarker for PDAC detection is widely used in clinical practice, yet it only yields a diagnostic sensitivity of 0.78 and a specificity of 0.77 [11]. The use of CA19–9 as a biomarker for PDAC diagnosis has been problematic as patients with symptom of jaundice and pancreatitis also have an elevated Ca19–9 level; additionally, patients with Lewis negative phenotype (a−/b-) have no expression of CA19–9 [7, 12–14]. Other potential molecular biomarkers (e.g., circulating tumor DNA, microRNA, or mutant TP53) as biomarkers for PDAC are still under investigation [15].

Saliva contains a broad spectrum of oral microbes [2]. Saliva microbiome analysis may holds promise for early PDAC detection [7]. A recently published study in China found *Streptococcus* and *Leptotrichina* were associated with a higher risk of PDAC while *Veillonella* and *Neisseria* were considered protective microbes that decrease the risk of PDAC. A population-based study of a European cohort found that oral *Candida* infection was associated with PDAC [16]. Similarly, an Asian cohort study conducted in Taiwan found that *Candida*-infected individuals have a higher risk of PDAC [17]. Mycobiome, especially pathogenic fungi, can stimulate PDAC by driving the complement cascade through mannose-binding lectin (MBL) activation [3, 18, 19], while the genera *Schizophyllum*, a member of the oral mycobiota, showed potential anti-cancer function [20]. Nevertheless, the role of oral mycobiota in the onset and progression of PDAC has not been comprehensively investigated [2]. Therefore, this study aimed to: (1) Determine the saliva mycobiota structure of PDAC using Internal Transcribed Spacer (ITS) ribosomal RNA sequencing; and (2) Select proper and specific mycobiota markers for PDAC detection.

## Methods

### Ethical consideration

This study was approved by the Institutional Review Board of the West China Hospital, Sichuan University (IRB Number: 20170420). The study was performed in accordance with the Helsinki Declaration and Rules of Good Clinical Practice. All participants signed written informed consent.

### Study design and participants

This study used a prospective design to consecutively recruit 54 adult patients who were suspected to have pancreatic tumor before biopsy or surgery. We excluded 5 patients with insufficient saliva samples (*n* = 5). Based on histopathological results, 34 patients were confirmed to have primary PDAC after biopsy and surgery. We recruited 35 healthy controls from the community as a comparison group. Healthy controls had normal liver and renal function, normal cardiopulmonary function, no history of cancer, and no viral infection. Exclusion criteria were: (1) A history of prior malignancy and chemotherapy or radiotherapy; (2) Metastatic PDAC or concurrent PDAC and other cancers; (3) A history of viral infection (i.e. hepatitis B virus, hepatitis C virus, human immunodeficiency virus); (4) Use of antibiotics (including oral, intravenous, or intramuscular) within 4 weeks before enrollment; and (5) Use of corticosteroids (nasal or inhaled) or other immunosuppressants [7]. A final sample of 69 included 34 patients with PDAC and 35 healthy controls.

### Phenotypic data collection

The demographic data (i.e., age, gender, and diseases history) were collected. Clinical data (i.e., cancer site, surgery types, and cancer stages by the American Joint Commission on Cancer (AJCC) Staging Manual–7th Edition [21]) were also collected. Symptom related to PDAC were assessed using a valid symptom checklist through patients’ self-report, including jaundice, dark brown urine, constipation, diarrhea, pale stools, pruritus, fatigue, pain, bloating, lack of appetite, nausea, vomiting, and disturbed sleep [7, 22]. The presence and absence of symptoms were reported by patients using “Yes” or “No.”

### Saliva Collection, DNA Extraction, and ITS1 gene sequencing

Saliva samples were obtained by trained medical personnel before surgery [7]. All the participants refrained from eating and drinking for at least 30 minutes before saliva collection. Approximately 3 mL of saliva were collected after it accumulated on the mouth floor from each participant by having them expectorate into specimen tubes. Fresh samples were placed in an ice bath and transported to the laboratory. Each sample was divided into 1.5 mL aliquots and immediately stored at − 20 °C for short-term storage, and at − 80 °C for long-term storage.

The Mag-Bind®Soil DNA Kit (M5635, Omega Bio-tek, Georgia, USA) was applied for total DNA extraction according to the manufacturer’s protocol. Primers ITS5F: 5′-GGAAGTAAAAGTCGTAACAAGG-3′ and ITS1R: 5′-GCTGCGTTCTTCATCGATGC-3 were used for the ITS1 fragment amplification. Library construction was implemented according to previous studies [23]. Then, sequencing was performed at paired-end 250 bp on the Illumina platform. The sequencing data were analyzed according to an integrated pipeline for fast amplicon data analysis [24].

### Statistical analysis

Descriptive statistical analyses were used to summarize means and standard deviation (SD) for the continuous variables; frequency and percentage (%) for the categorical variables. Fisher’s exact tests were performed for categorical variables. T-test and Mann-Whitney U test were used for continuous variables. Statistical significance was established at *P* < 0.05 with a 95% confidence interval (CI). In the mycobiota-related data analysis, *P*-values were corrected for false discovery rate (FDR) (statistical differences established at FDR < 0.05) [24]. The statistical analyses were performed using the SPSS (v23.0, SAGE IBM, Armonk, NY, United States) and R software, version 4.0.3.

To estimate PDAC risk prediction, logistic regressions were performed to analyze the association of specific taxa with clinical covariates. To prevent the occurrence of false-negative diagnosis, only the top 20 taxa (OTUs abundance) and the mycobiota associated with PDAC were reported. To make the values comparable, the OTU values were converted into a normalized z-score. The Z-score was used to standardize QUI (Z = (x-mean value)/standard deviation, Z-score = − 1 ~ + 1). Odds ratio with 95% CIs were estimated.

## Results

### Mycobiota profile

**Fig. 1:**
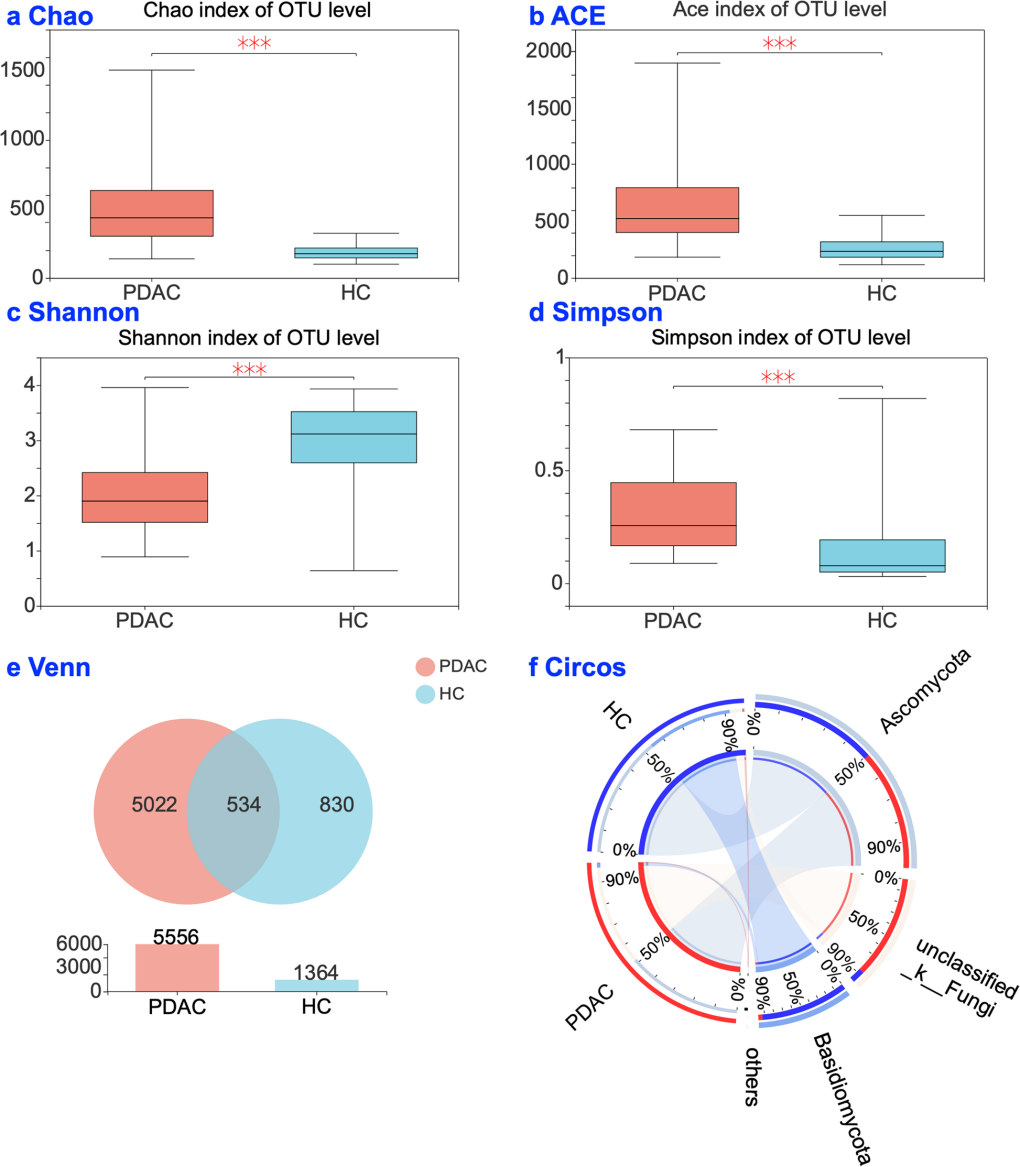
Mycobiota profiles. **a** Chao index at OTU level. **b** ACE index at OTU level. **c** Shannon index at OTU level. **d** Simpson index at OTU level. **e** Venn diagram at OTU level, which showing shared and unique operational taxonomic units (OTUs) between the two groups. f Circos analysis on the structure of mycobiota at phylum level. PDAC: pancreatic ductal adenocarcinoma patients (*n =* 34); HC: healthy controls (*n =* 35). * *P* < 0.05, **P < 0.01, ***P < 0.001

We examined mycobiota profile of 69 samples, including 34 patients with PDAC and 35 Healthy controls. Table 1 provides more information of the samples. We filtered 4,490,598 qualified reads; 1,485,984 reads were randomly chosen (69 × 21,536 reads/sample, the minimum number of reads/sample). Finally, 21,536 operational taxonomic units (OTUs) and an average Good’s coverage of 99.6% of each sample were obtained for further analysis (Fig. S1). As Fig. 1 displayed, compared with the healthy controls, the PDACs group had significant increases in fungal abundance estimated by the Chao index (Fig. 1a, *P* < 0.001) and ACE index (Fig. 1b, *P* < 0.001). A significant decrease was found in fungal diversity estimated by Shannon index (Fig. 1c, *P* < 0.001) and Simpson index (Fig. 1d, *P* < 0.001). As shown in Fig. 1e, PDAC and healthy groups shared 534 species, PDAC patients had 5022 unique OTUs, and healthy controls had 830 unique OTUs. Circos analysis at the phylum level (Fig. 1f) indicated that there were differences in the community composition of *Basidiomycota* and *unclassified fungi* between PDAC patients and healthy controls.

**Table 1:** Demographic and clinical characteristics

Variables	PDAC ^a^ (*n =* 34)	HC ^b^ (*n =* 35)
**Age**, Mean ± SD^d^	60.97 ± 11.9	60.51 ± 6.34
**Gender**, n *(%)*		
Male	19 (55.9)	20 (57.1)
Female	15 (44.1)	15 (42.9)
**Smoking**, n *(%)*	14 (41.2)	12 (34.3)
**Drinking**, n *(%)*	13 (38.2)	14 (40)
**Jaundice**, n *(%)*	15 (44.1)	–
**Dark brown urine**, n *(%)*	16 (47.1)	–
**Primary cancer location**, n *(%)*		
Head of Pancreas	26(78.8)	–
Body and Tail of Pancreas	7(21.2)	–
**Types of Surgery**, n *(%)*	n *(%)*	–
Pancreaticoduodenectomy	13 (38.2)	–
Distal pancreatectomy	4 (11.8)	–
Palliative intervention techniques	17 (50.0)	–
**Tumor grading / AJCC staging**^**c**^, n *(%)*		
I-IIB	16(47.1)	–
III-IV	18(52.9)	–

^a^ *PDAC* Pancreatic ductal adenocarcinoma patients;

^b^ *HC* Healthy controls

^c^ *AJCC* American Joint Commission on Cancer

^d^ *SD* Standard deviation

**Fig. 2:**
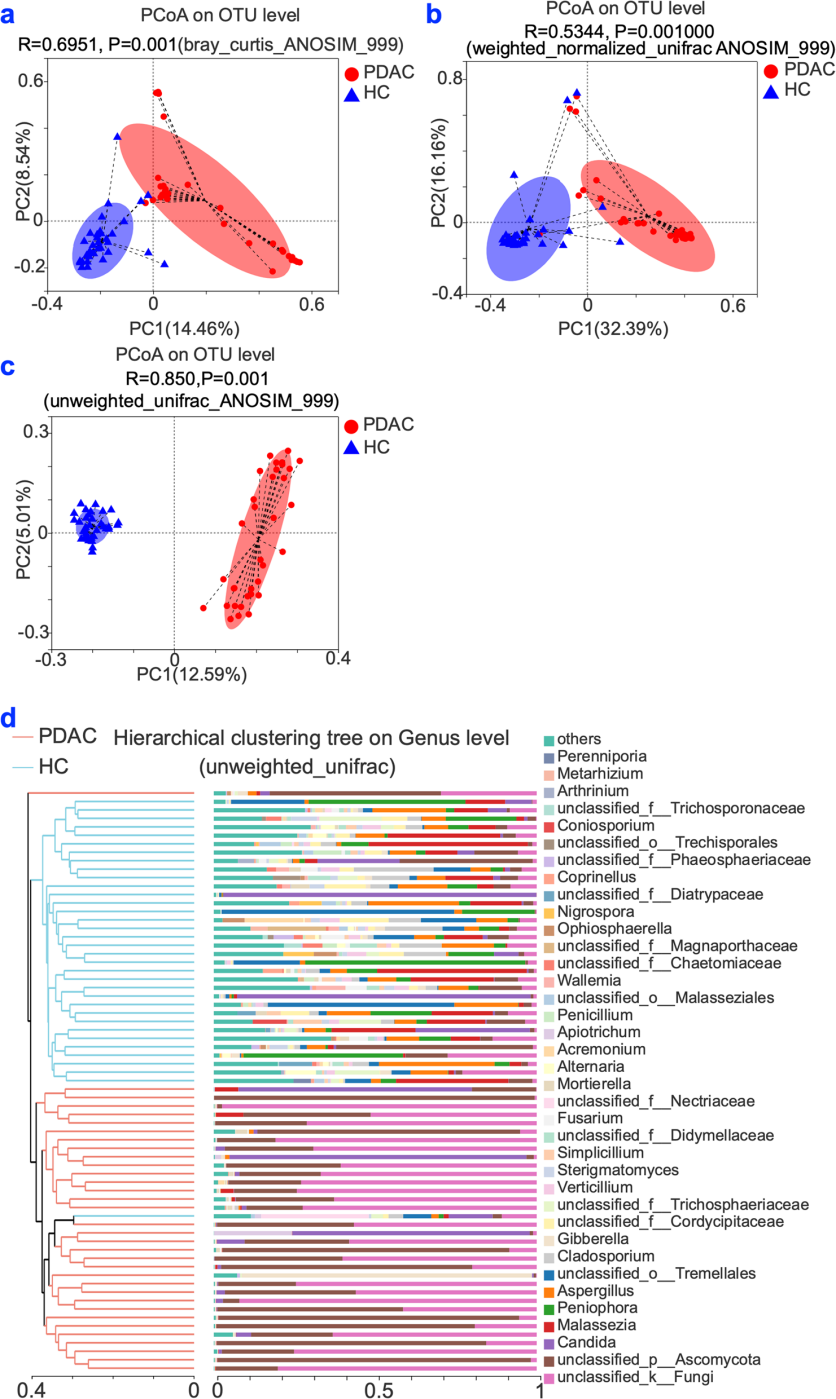
Beta-diversity and Hierarchical clustering. **a-c** PCoA analysis. *P*-values were defined from 999 permutations during the analysis of similarity test (ANOSIM). **d** Hierarchical clustering at genus level. PDAC: pancreatic ductal adenocarcinoma patients (*n =* 34); HC: healthy controls (*n =* 35)

The Principal Coordinates Analysis (PCoA) results indicated that the fungal communities from the PDAC patients and healthy controls were partly separated based on the Bray–Curtis dissimilarity matrix (Fig. 2a, analysis of similarity test (ANOSIM): *P*-value = 0.001), weighted normalized unifrac distance (Fig. 2b, ANOSIM: *P*-value = 0.001), and unweighted unifrac distance (Fig. 2c, ANOSIM: *P*-value = 0.001). Similar results were also found in hierarchical clustering ITS1 sequences at genus level (Fig. 2d).

### Fungal taxonomic alterations in PDAC

**Fig. 3:**
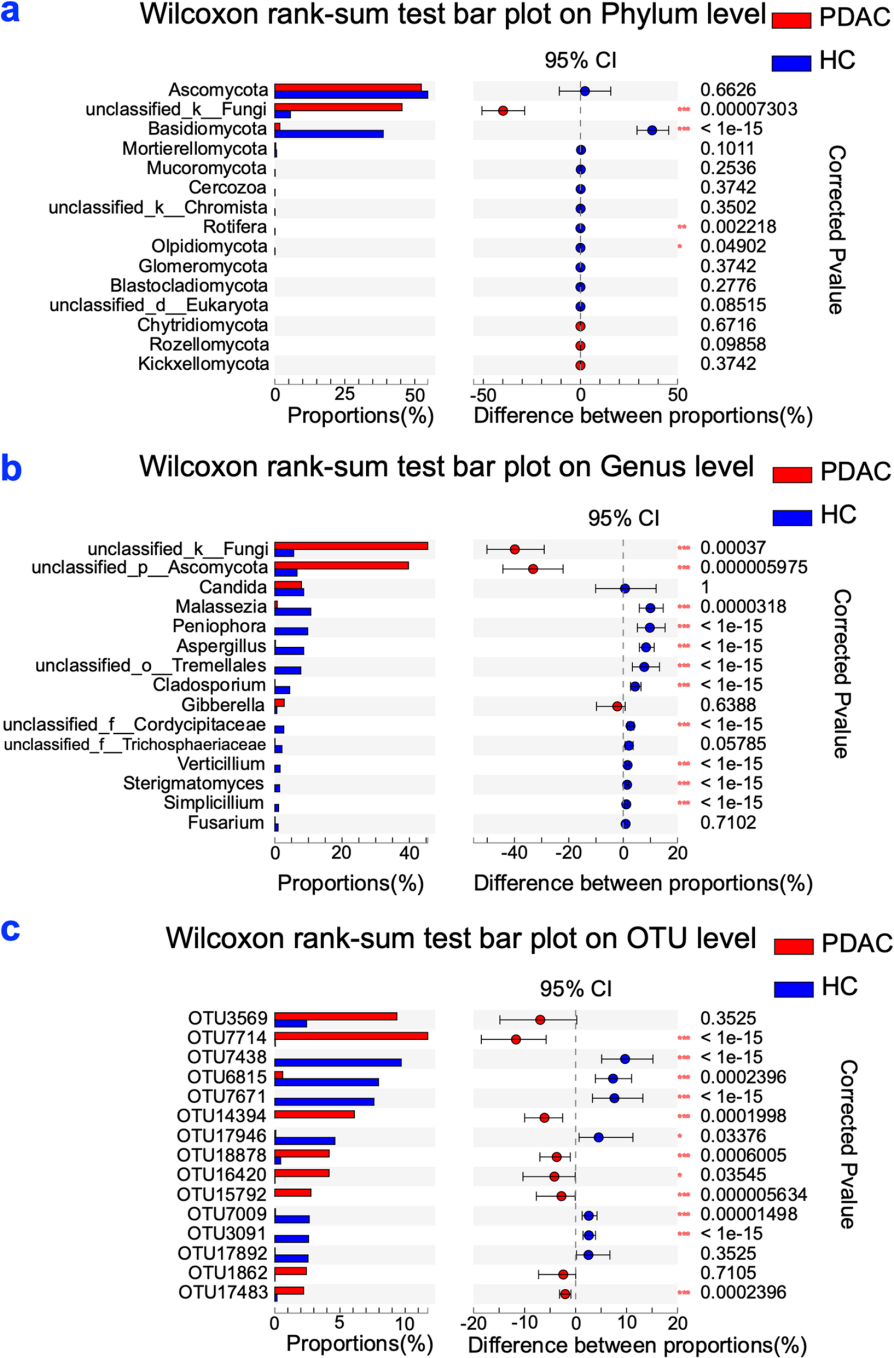
Fungal taxonomic alterations. **a** Taxa differences at phylum level. **b** Taxa differences at genus level. **c** Taxa differences at OTU level. PDACs: pancreatic ductal adenocarcinoma patients (*n =* 34); HCs: healthy controls (*n =* 35); *, corrected *P*-value < 0.05, **, corrected *P*-value < 0.01, ***, corrected *P*-value < 0.001

To evaluate fungal taxonomic alterations, we examined the main fungal taxa differences between PDAC and healthy control groups using the Wilcoxon rank-sum test at the phylum level (Fig. S2a, Fig. 3a), genus level (Fig. S2b, Fig. 3b) and OTU level (Fig. S2C, Fig. 3c). Significant differences in specific fungal taxa were found between PDAC and healthy control groups; these fungal taxa included *unclassifed_k_fungi*, *Basidiomycota*, *Unclassifed_p_Ascomycota*, *Aspergillus*, and *Cladosporium*, etc.

### Saliva mycobiota for PDAC prediction

**Fig. 4:**
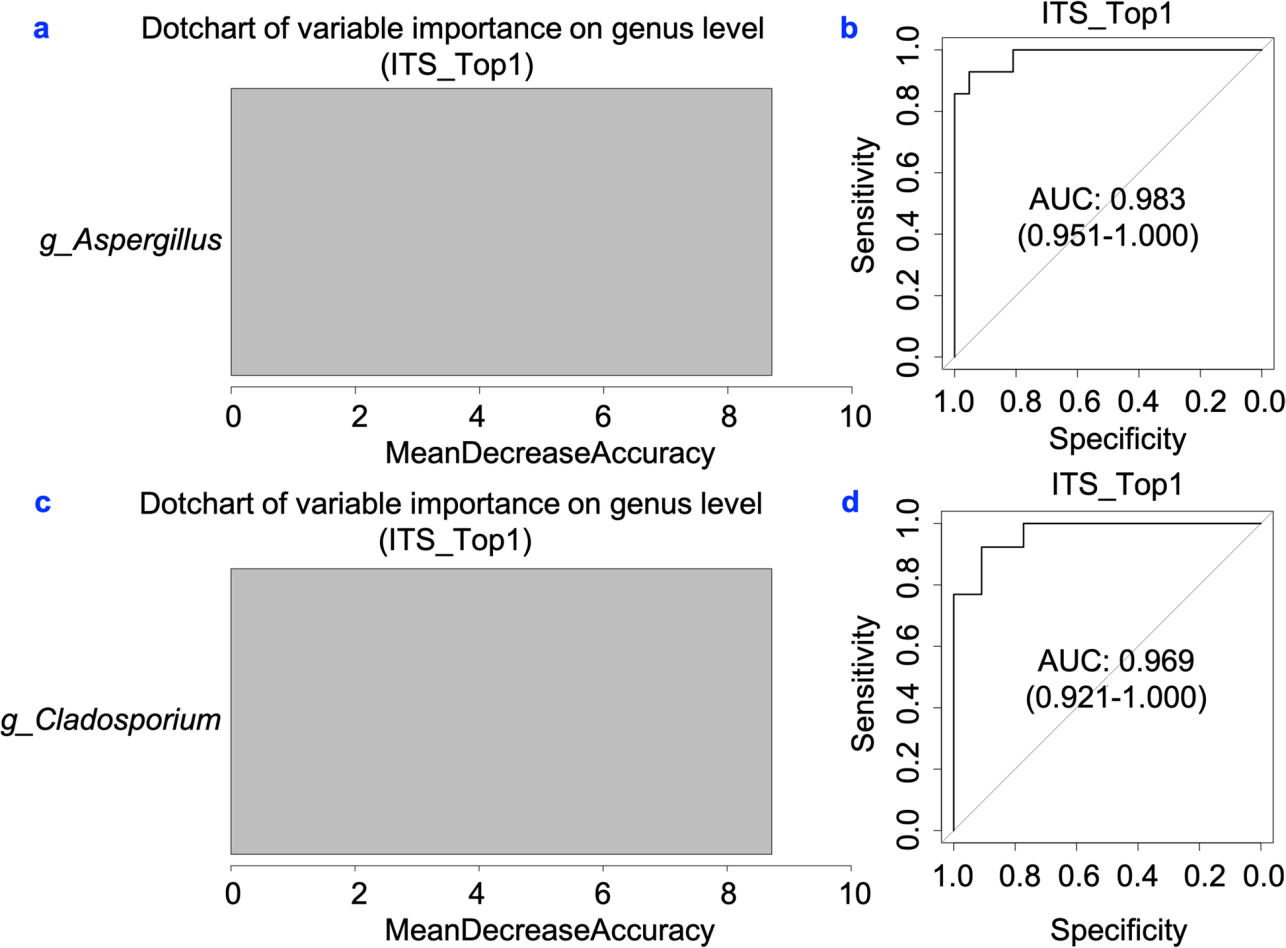
Classification power of specific fungal markers associated with PDAC by ROC analysis. **a, c** random forest analysis being used to explore the feature contributions (specific fungal markers). **b, d** ROC curves based on the random forest model. PDAC: pancreatic ductal adenocarcinoma patients (*n =* 34); HC: healthy controls (*n =* 35); ROC, receiving operational curve; AUC, area under curve; the number of participants in each group

To explore the role of saliva mycobiota to predict PDAC, we randomly divided PDAC patients and healthy controls into two equal parts. Random forest construction and Receiver operating characteristic (ROC) analysis were performed independently for each randomly divided part. The abundance of genus *Aspergillus* alone was able to distinguish PDAC patients from healthy controls (AUC = 0.983, 95% CI 0.951–1.000; Fig. 4a and Fig. 4b). Similar results were also found in genus *Cladosporium* (AUC = 0.969, 95% CI 0.921–1.000; Fig. 4c and Fig. 4d).

### Fungal functional prediction analysis

**Fig. 5:**
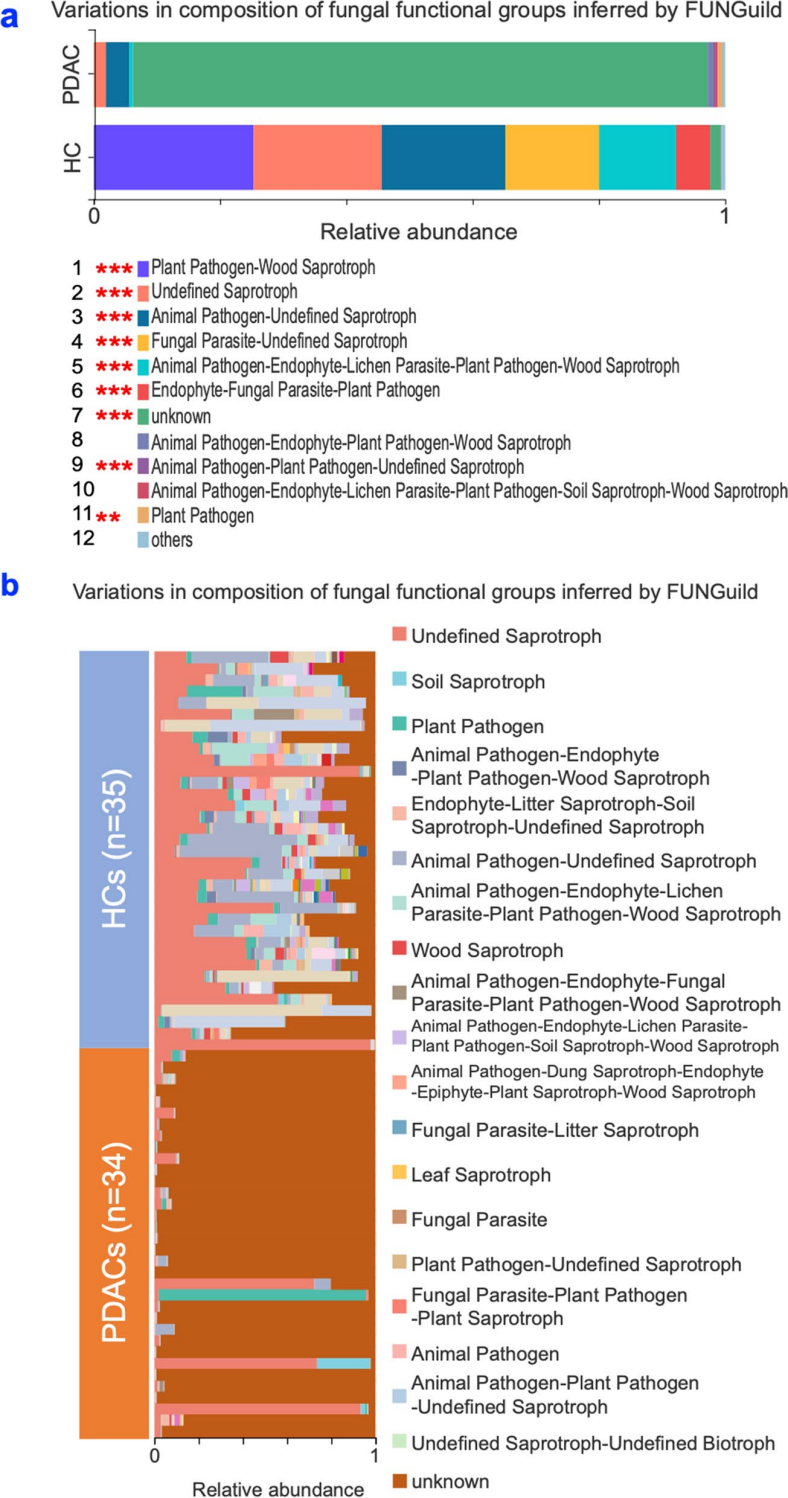
FUNGuild analysis for the functional prediction of saliva mycobiota. **a** The relative abundance between two groups assigned by FUNGuild for fungal communities. **b** The relative abundance of each sample assigned by FUNGuild for fungal communities. PDAC: pancreatic ductal adenocarcinoma patients (*n =* 34); HC: healthy controls (*n =* 35); *, corrected *P*-value < 0.05, **, corrected *P*-value < 0.01, ***, corrected *P*-value < 0.001

Fungal function profiles between PDAC and healthy control groups were analyzed via FUNGuild database using Kruskal-Wallis H tests. Figure 5a demonstrated that there was a significant difference between PDAC patients and healthy controls. Among the 12 main combinations of categories, 10 significant differences were observed, including “Undefined Saprotroph”, “Animal Pathogen-Undefined Saprotroph”, and “unknown” category. PDAC patients had significantly higher abundance of the “unknown” category than the healthy controls. Meanwhile, PDAC patients had significantly lower abundance in the other 9 categories, such as the “undefined Saprotroph” (Fig. 5b) than the healthy controls (Table S1).

### Mycobiota profile and symptoms related to PDAC

We explored the associations of mycobiota with symptoms related to PDAC. Table 2 presented differences in fungal abundance between the PDAC patients with symptoms and without symptoms. Patient who reported jaundice had a greater abundance of *g__unclassified_p__Ascomycota* compared to those without jaundice (52.0 ± 31.9 vs. 30.0 ± 25.7, *P* = 0.037). Patients who reported dark brown urine presented a greater abundance in *g__unclassified_p__Ascomycota* compared to those without dark brown urine (51.1 ± 31.1 vs. 29.5 ± 26.2 *P* = 0.037). No significant fungal differences were observed among patients with different cancer staging based on American Joint Commission on cancer staging criteria and types of surgery (Table 2).

**Table 2:** Fungal abundance and phenotypic characteristics of PDAC^a^

Variables	Mycobiota	Mean ± SD^**b**^	Mean ± SD^**b**^	***P***-values	Effect size (95% CI)^**d**^
		Yes ^c^	No ^c^		
Jaundice	g__unclassified_p__Ascomycota	52.0 ± 31.9	30.0 ± `25.7	**0.037**	22.0 (4.41 ~ 41.7)
Dark brown urine	g__unclassified_p__Ascomycota	51.1 ± 31.1	29.5 ± 26.2	**0.037**	21.6 (2.08 ~ 38.6)
AJCC staging^e^		Stage I-IIB	Stage III-IV		
	g__unclassified_k__Fungi	47.0 ± 34.8	43.6 ± 29.3	0.759	3.45 (−19. ~ 26.0)
	g__unclassified_p__Ascomycota	33.4 ± 29.3	46.8 ± 30.6	0.203	−13.0 (−34. ~ 7.58)
	g__Malassezia	0.77 ± 1.87	0.69 ± 2.10	0.907	0.08 (−1.3 ~ 1.47)
Types of Surgery^f^		Resectable PDAC	Unresectable PDAC		
	g__unclassified_k__Fungi	51.1 ± 32.8	39.7 ± 30.8	0.256	11.3 (−9.6 ~ 32.6)
	g__unclassified_p__Ascomycota	34.0 ± 27.1	45.4 ± 32.9	0.228	−11.37 (29.81 ~ 7.60)
	g__Malassezia	0.81 ± 1.92	0.65 ± 2.04	0.705	0.15 (−1.1 ~ 1.52)

^a^ *PDAC* Pancreatic ductal adenocarcinoma patients (*n =* 34)

^b^ *SD* Standard deviation

^c^ *Yes* Patients with a given symptom; No: patients without a given symptoms

^d^ *CI* Confidence interval

^e^ Patients diagnosed with stage I-IIB (*n =* 16); patients diagnosed with stage III-IV (*n =* 18)

^f^ Patients had resectable PDAC (Pancreaticoduodenectomy or Distal pancreatectomy) (*n =* 17); Patients had unresectable PDAC (Palliative intervention techniques) (*n =* 17)

### Logistic regression for mycobiota profile

We performed logistic regressions to quantify the effects of selected fungi abundances (normalized z-score) on PDAC risk. As shown in Table 3, compared with healthy controls, carriage of *g__unclassified_k__Fungi* (OR = 1.359, 95%CI: 1.113 ~ 1.66, *P* = 0.003) and *g__unclassified_p__Ascomycota* (OR = 1.260, 95%CI: 1.086 ~ 1.463, *P* = 0.002) were associated with a higher risk of PDAC. With each increase of abundance of *g__unclassified_k__Fungi* and *g__unclassified_p__Ascomycota* in PDAC patients, the risk of pancreatic cancer increased by 1.359 odds and 1.260 odds, respectively. No oral fungi protective factors of PDAC were found.

**Table 3:** Oral fungi distribution and risk of PDAC

	Odds ratio (95%CI^a^)	*P*-values	Hosmer–Lemeshow test
Healthy Controls (*n =* 35)	Reference		Chi-square value = 9.467, Degree of freedom = 8, *P*-value = 0.304
PDAC^b^ (*n =* 34)			
Age	1.637(0.463 ~ 5.791)	0.445	
Sex	0.301(0.032 ~ 2.799)	0.291	
*g__unclassified_k__Fungi*	1.359(1.113 ~ 1.66)	**0.003**	
*g__unclassified_p__Ascomycota*	1.260(1.086 ~ 1.463)	**0.002**	
*g__Candida*	1.181(0.998 ~ 1.397)	0.052	
*g__Malassezia*	0.263(0.062 ~ 1.111)	0.069	

^a^ *CI* Confidence interval

^b^ *PDACs* Pancreatic ductal adenocarcinoma patients

## Discussion

Recent studies indicated the role of gut mycobiota in pancreatic, oesophageal, and colonic oncogenesis [3, 19, 25, 26]. Meanwhile, emerging evidence highlighted the importance of fungi in gastrointestinal disease and cancers [27–29]. Jain et al. discovered that *Debaryomyces* inhabited tissue inflammation in Crohn’s disease which may lead to dysregulated mucosal healing [30]. Zhong et al. found gastric cancer-associated mycobiome dysbiosis characterized by significant increase of *C. albicans* and significant decrease of the diversity and richness of fungi in the stomach of patients with gastric cancer [31]. Together with our findings, these recent advancements not only support that certain commensal fungi might be related to gastrointestinal cancers, [32] but also highlight the feasibility of mycobiota-driven approaches to identify patients at risk of gastrointestinal disease and cancers.

Studies using animal models found a ~ 3000-fold fungal increase in PDAC [3]. Findings of our study extended the knowledge of alpha-diversity of mycobiota on PDAC by demonstrating a significant increase in fungal abundance and significant decrease in fungal diversity (Fig. 1) in PDAC patients compared with the healthy controls. Prior studies found fecal microbiota consistently had high Chao index and low Shannon index in PDAC patients [33]. Similar alpha diversity profiles of PDAC patients were found using oral mycobiota, oral microbiota and fecal microbiota [7, 20]. Fecal sample-based measurements have been used for the studies on the microbiome of pancreatic cancer [3, 33, 34], saliva sample-based methods were more convenient and more controllable for the quality of sample during sample collection. Yet, decreased abundance of *Malassezia* in PDAC patients was found in our study using saliva sample method but was increased in the study by Aykut [3] using pancreatic tissue and normal tissue. A high relative abundance of *Malassezia* demonstrated association with favorable survival in oral squamous cell carcinoma (OSCC) patients. Mohamed et al., found that the fungal genus Malassezia could be a putative prognostic biomarker and therapeutic target for OSCC [35]. The conflicting findings may be because of different types of samples. Future studies should focus on the comparison of different samples, e.g., saliva vs. tissue. In addition, geographic, race and diet differences may be the other factors for the conflicting findings [18, 36, 37]. For instance, patients in Aykut’ s study were enrolled from the NYU Langone Medical Center, New York, United States, where Caucasians are the main race [3]. Participants in our study were from the Sichuan Province, China where all people prefer a rice-based diet adding loads of herbs and spices [38, 39]. Future studies should evaluate the effects of geographical location, race and diet on mycobiota analysis [40].

In terms of mycobiota composition and its PDAC prediction ability, we found that *Basidiomycota* and *Unclassifed_p_Ascomycota* had a significant increase in PDAC patients. Aykut et al. found only *M. globosa* accelerated the growth of pancreatic tissue, whereas the other taxa, such as *Aspergillus*, *Candida*, and *Saccharomyces*, had no effect on pancreatic tissue using animal and physiologic models [3]. Our study found that the PDACs patients had a significant decrease in *Aspergillus* and *Cladosporium* compared to healthy controls. Notably, *Aspergillus* and *Cladosporium* achieved a high classification power between PDAC patients and healthy controls. This finding is promising in that *Aspergillus* and *Cladosporium* may serve as noninvasive biomarkers for PDAC detection and screen.

Symptoms are the indicators of abnormal body functions that often indicate earlier physiological changes when objective measures are not attainable [22, 41, 42]. A recent study demonstrated that symptom patterns and scores were able to differentiate patients from Stage I and Stage IV PDAC cancer and patients with pancreatic non-cancer tumors [22]. Our study was the first to investigate the symptom-mycobiota interaction. We found that symptomatic PDAC patients had different mycobiota profiles than asymptomatic PDAC patients. Patients reporting jaundice had significant greater abundance of *g_unclassified_p_Ascomycota* compared to those without jaundice. Patients with dark brown urine have significant higher abundance in *g_unclassified_p_Ascomycota* compared to those without dark brown urine. Aykut [3] et al found that *Basidiomycota* and *Ascomycota* were the only phyla discovered in pancreatic tissue, whereas other fungal phyla were additionally discovered in the normal tissue. Symptomatic PDAC patients had different mycobiota profiles than asymptomatic PDAC patients. Given limitations of current pancreatic cancer screening (e.g. CT, MRI, ultrasound endoscope), symptom evaluation coupled with oral mycobiota may be an efficient initial screening tool for surveillance of high-risk individuals followed by diagnostic tests.

Resectable PDACs (e.g., Pancreaticoduodenectomy or Distal pancreatectomy) usually have a superior prognosis with surgical operation than unresectable PDAC (i.e., Palliative interventions). While oral microbiota was found to be able to discriminate resectable PDAC and unresectable PDAC [7], our study was the first to evaluate the oral mycobiota feature of PDAC patients with different surgery types and AJCC stages (I-IIB vs. III-IV) (Table 2). No mycobiota was found to be able to distinguish patients from different AJCC stages (i.e., I-IIB vs. III-IV) and surgery types (i.e., resectable PDAC (Pancreaticoduodenectomy or Distal pancreatectomy) and unresectable PDAC (Palliative interventions). However, due to the limitations of our sample size, future research with a large sample is warranted.

The FUNGuild database was applied to predict the nutritional and functional groups of the mycobiota [43]. It can be only classified into three main trophic modes: phagotroph, symbiotroph, saprotroph. Within these trophic modes, 12 categories were further designated, namely: animal pathogens, arbuscular mycorrhizal fungi, ectomycorrhizal fungi, ericoid mycorrhizal fungi, foliar endophytes, lichenicolous fungi, lichenized fungi, mycoparasites, plant pathogens, undefined root endophytes, undefined saprotrophs, and wood saprotrophs [44]. Plant Pathogen (7 times), Animal Pathogen (5 times), Endophyte (4 times), Wood Saprotroph (4 times), and Undefined Saprotroph (4 times) were the major categories found in our study. In addition, we observed that “unknown” were dominated in PDAC patients, while the other 9 categories, such as the “Undefined Saprotroph” were in healthy controls. All these functional changes of oral fungal communities between PDAC patients and healthy controls suggest that mycobiota might affect the onset and progress of pancreatic disease.

In Fig. 5, mycobiota variations were mainly “unknown” in PDAC patients. Many research studies have focused on microbiota rather than mycobiota [45, 46]. Thus, the phenomenon that mycobiota variations were unknown in Fig. 5 also reflected the lack of research to identify the mycobiota variation. With more research focusing on mycobiota, more and more new fungi would be discovered in future research [47].

Findings of our study should be considered in the light of study limitations. First, our sample size was relatively small, although rarefaction curve (Fig. S1) indicated that the sequencing depth of each subgroup was sufficient. Second, we only used ITS1 sequencing to analyze mycobiota. Because of the limitations of FUNGuild [43], only preliminary functional prediction was performed in our study. Metagenomic detection should be considered in further study to better validate the function of the oral mycobiota from different populations. Third, it was not feasible for us to exclude participants with dental disease since many of our participants were unable to give accurate history of dental disease, and no medical records or documents were available for us to verify the dental disease status. Professional dentist examinations should be considered in future studies. One strength of this study was that we compared pancreatic cancer patients and healthy controls to find the gradual change trend of mycobiota distribution.

In conclusion, saliva mycobiota were able to distinguish PDAC patients from the healthy controls. A higher abundance of *Basidiomycota* and *Unclassifed_p_Ascomycota* was associated with an increased risk of PDAC. *Aspergillus* and *Cladosporium* achieved high classification powers to discriminate PDAC patients and healthy controls. It is worth noting that the rapid, inexpensive tests of ITS1 sequencing of mycobiota and PCR detection of potential fungal biomarkers, such as *Aspergillus* and *Cladosporium* make it promising for clinical practice to use oral microbes, including mycobiota, for PDAC early detection and prevention.

## Supplementary Information

**Additional file 1.**

**Additional file 2.**

## Data Availability

The datasets generated for this study can be found in the NCBI’s Sequence Read Archive under BioProject ID PRJNA 837102, sample accession SAMN28196229- SAMN28196297. The publicly available link for the data is listed below: https://dataview.ncbi.nlm.nih.gov/object/PRJNA837102?reviewer=pstmdg7vm4hnlbms1k5bbisejc. Alternative publicly available link for the data along with its accession number (Password: fcde): https://pan.baidu.com/s/1yiLnIY28DDhF2tmzhNucFA
