# Supplementary material for: Oral mycobiota and pancreatic ductal adenocarcinoma
Source: BMC Cancer. 2022 Dec 2;22:1251. doi: 10.1186/s12885-022-10329-5 (PMC9716801; doi:10.1186/s12885-022-10329-5)
Supplement: Supplementary file 1 — Additional file 1. [file 12885_2022_10329_MOESM1_ESM.docx]

**Supplementary Figure legend.**

**Figure S1**. **The rarefaction curves analysis**.

PDAC: pancreatic ductal adenocarcinoma patients (n=34); HC: healthy controls (n=35)

**Figure S2. Compositional differences in the salivary mycobiota.**

(a) at phylum level.

(b) at genus level.

(c) at OTU level.

PDAC: pancreatic ductal adenocarcinoma patients (n=34); HC: healthy controls (n=35).
