# Supplementary material for: Oral mycobiota and pancreatic ductal adenocarcinoma
Source: BMC Cancer. 2022 Dec 2;22:1251. doi: 10.1186/s12885-022-10329-5 (PMC9716801; doi:10.1186/s12885-022-10329-5)
Supplement: Supplementary file 2 — Additional file 2. [file 12885_2022_10329_MOESM2_ESM.docx]

**Supplementary Table.**

**Table S1 FUNGuild analysis for the fungal functional prediction using Kruskal-Wallis H tests**

| **No.** | **FUNGuild** | **PDAC** ^1^ **(n=34)** | **HC** ^2^ **(n=35)** | Corrected P-value |
| --- | --- | --- | --- | --- |
| 1 | Plant Pathogen-Wood Saprotroph | 0.79+2.11 | 2163.83+3333.58 | **<0.001** |
| 2 | Undefined Saprotroph | 1903.15+4890.05 | 6235.6+4661.42 | **<0.001** |
| 3 | Animal Pathogen-Undefined Saprotroph | 161.35+420.53 | 2311.46+2919.12 | **<0.001** |
| 4 | Fungal Parasite-Undefined Saprotroph | 2.24+2.83 | 1765.8+3349.32 | **<0.001** |
| 5 | Animal Pathogen-Endophyte-Lichen Parasite-Plant Pathogen-Wood Saprotroph | 12.97+43.97 | 955+1205.07 | **<0.001** |
| 6 | Endophyte-Fungal Parasite-Plant Pathogen | 0.12+0.41 | 342.11+421.91 | **<0.001** |
| 7 | unknown | 18406.62+6326.21 | 3966.8+3070.46 | **<0.001** |
| 8 | Animal Pathogen-Endophyte-Plant Pathogen-Wood Saprotroph | 9.03+13.04 | 186.69+408.18 | 0.515 |
| 9 | Animal Pathogen-Plant Pathogen-Undefined Saprotroph | 4.62+6.6 | 351.71+593.49 | **<0.001** |
| 10 | Animal Pathogen-Endophyte-Lichen Parasite-Plant Pathogen-Soil Saprotroph-Wood Saprotroph | 27.06+35.14 | 216.8+428.42151 | 0.602 |
| 11 | Plant Pathogen | 656.88+3455.82 | 523.17+916.58 | **0.003** |

^1^ PDAC: pancreatic ductal adenocarcinoma patients; ^2^ HC: healthy controls.
